# Neural network-assisted single-molecule localization microscopy with a weak-affinity protein tag

**DOI:** 10.1016/j.bpr.2023.100123

**Published:** 2023-08-18

**Authors:** Soohyen Jang, Kaarjel K. Narayanasamy, Johanna V. Rahm, Alon Saguy, Julian Kompa, Marina S. Dietz, Kai Johnsson, Yoav Shechtman, Mike Heilemann

**Affiliations:** 1 Institute of Physical and Theoretical Chemistry, Johann Wolfgang Goethe-University, Frankfurt am Main, Germany; 2 Institute of Physical and Theoretical Chemistry, IMPRS on Cellular Biophysics, Johann Wolfgang Goethe-University, Frankfurt am Main, Germany; 3 Department of Functional Neuroanatomy, Institute for Anatomy and Cell Biology, Heidelberg University, Heidelberg, Germany; 4 Department of Biomedical Engineering, Technion – Israel Institute of Technology, Haifa, Israel; 5 Department of Chemical Biology, Max Planck Institute for Medical Research, Heidelberg, Germany

## Abstract

Single-molecule localization microscopy achieves nanometer spatial resolution by localizing single fluorophores separated in space and time. A major challenge of single-molecule localization microscopy is the long acquisition time, leading to low throughput, as well as to a poor temporal resolution that limits its use to visualize the dynamics of cellular structures in live cells. Another challenge is photobleaching, which reduces information density over time and limits throughput and the available observation time in live-cell applications. To address both challenges, we combine two concepts: first, we integrate the neural network DeepSTORM to predict super-resolution images from high-density imaging data, which increases acquisition speed. Second, we employ a direct protein label, HaloTag7, in combination with exchangeable ligands (xHTLs), for fluorescence labeling. This labeling method bypasses photobleaching by providing a constant signal over time and is compatible with live-cell imaging. The combination of both a neural network and a weak-affinity protein label reduced the acquisition time up to ∼25-fold. Furthermore, we demonstrate live-cell imaging with increased temporal resolution, and capture the dynamics of the endoplasmic reticulum over extended time without signal loss.

## Why it matters

Single-molecule localization microscopy (SMLM) allows visualization of cellular structures at the nanoscale. However, SMLM requires low-molecular-density images, which results in long acquisition times and thus low throughput. This further translates into a poor temporal resolution, limiting its application to visualize structural dynamics in live cells. Another challenge is the loss of fluorescence signal because of photobleaching, reducing the information density over time and limiting the accessible observation time for live-cell imaging.

Here, we reduce the acquisition time ∼25-fold using a neural network, and implement a live-cell compatible protein tag that provides a constantly “renewed” fluorescence signal. We demonstrate multitarget imaging, increase the temporal resolution in live-cell imaging, and capture the dynamics of the endoplasmic reticulum over extended time.

## Introduction

A key element for understanding cellular architecture lies in the ability to visualize biomolecules with near-molecular spatial resolution using light microscopy (1). For most super-resolution techniques, a compromise has to be made between temporal versus spatial resolution. Imaging methods such as stimulated emission depletion (STED) (2,3) and structured illumination microscopy (4,5) are able to capture fast biological dynamics in live cells. In contrast, single-molecule localization microscopy (SMLM) methods (6), such as (fluorescence) photoactivated localization microscopy ((F)PALM) (7,8), (*direct*) stochastic optical reconstruction microscopy (*(d)*STORM) (9,10), and (DNA-) point accumulation in nanoscale topography (PAINT) (11,12), can achieve a spatial resolution in a range of only a few nanometers (13), and even below, as recently demonstrated by MINFLUX (14) and Exchange-PAINT (15). These methods are dependent on the accumulation of spatially isolated fluorescence emitters over time to resolve a structure. Due to its working principle, SMLM methods suffer from low temporal resolution, which limits the throughput and the visualization of fast processes such as dynamics in live cells. Efforts have been made to speed-up SMLM imaging using modified DNA probes (16) and imaging buffer compositions that promote shorter probe-to-target association rates (17,18). Other exciting developments are deep learning-assisted computational tools that successfully demonstrated a reduction of the acquisition time. This was achieved either by predicting super-resolved SMLM images from sparse imaging data (19,20) or from high-density imaging data (21,22,23,24). Recently, the neural network (NN) DeepSTORM (21) was used to accelerate DNA-PAINT image generation (25).

Another challenge is the loss of fluorescence signal due to photobleaching. Noncovalent fluorophore labels offer a significant advantage in various super-resolution imaging modes compared with permanently bound dyes and fluorescent proteins (26). Here, the imaging buffer containing the probe provides constant replenishment of target protein labeling, minimizing the effect of photobleaching. Imaging of live-cell dynamics can essentially proceed indefinitely given a large enough reservoir of dyes in the imaging buffer and good cell viability. This concept has been successfully implemented in multicolor, 3D, and live-cell STED microscopy using PAINT and DNA-PAINT labels (27,28). An exciting new development is direct protein labeling using the HaloTag as a protein tag in combination with exchangeable ligands for fluorescence labeling (xHTLs), which reversibly and transiently bind to HaloTag (29). This method allows direct labeling of target proteins, circumventing the need of secondary labels, provides a constant signal over time, is compatible with live-cell imaging, and was shown to be compatible with various super-resolution imaging modalities (29,30).

In this report, we synergize direct protein labeling using two HaloTag variants with exchangeable HaloTag ligands (xHTLs) (29), high-density imaging and image prediction with DeepSTORM (21). This combination allowed us to reduce the acquisition time significantly. Furthermore, we demonstrate multicolor imaging and live-cell imaging with increased temporal resolution, and capture the dynamics of the endoplasmic reticulum (ER) over extended time.

## Materials and methods

### Cell culture and sample preparation

Stable cell lines were generated using the Flp-IN T-REx system (Thermo Fisher Scientific, Waltham, Massachusetts). In brief, U-2 OS Flp-IN T-REx were cotransfected with pOG44 and pcDNA5/FRT/TO-GOI plasmids (10:1) and selected with hygromycin B (50 *μ*g/mL, Thermo Fisher Scientific, Waltham, Massachusetts) following a published protocol (31). Thus, TOM20 tagged with dHaloTag 7 (dHT7) is expressed on the outer membrane of mitochondria and CalR-KDEL tagged with HaloTag 7 (HT7) is expressed on ER, separated by a self-cleaving peptide sequence (T2A) (32), as described in Kompa et al. (29). CRISPR-Cas9-mediated knockin U-2 OS cells (33) that express vimentin-HT7 were a kind gift from Prof. Stefan Jakobs (MPI for Multidisciplinary Sciences, Göttingen, Germany).

U-2 OS stable cell lines were cultured in T-75 flasks (Greiner, Kremsmünster, Austria) at 37°C and 5% CO_2_ in Dulbecco’s modified Eagle’s medium (DMEM)/F-12 (Gibco, Thermo Fisher Scientific, Waltham, Massachusetts) containing 10% (v/v) fetal bovine serum (Gibco, Billings, Montana), 1% (w/v) penicillin-streptomycin (Gibco, Thermo Fisher Scientific, Waltham, Massachusetts), and 1% (v/v) GlutaMAX (Gibco, Billings, Montana). Two days before imaging, 2 × 10^4^ U-2 OS cells expressing the protein of interest were seeded on a fibronectin-coated 8-well chamber slide (Sarstedt, Nümbrecht, Germany) and the expression of pcDNA5/FRT/TO HT7-tagged protein was induced with 100–250 *μ*g/mL of doxycycline (Alfa Aesar, Ward Hill, Massachusetts).

### PAINT imaging

For PAINT imaging in fixed cells, U-2 OS cells expressing the protein of interest with an HT7 or dHT7 were fixed. For vimentin structure, cells were fixed using 4% formaldehyde (FA) (Thermo Scientific, Waltham, Massachusetts) in 1× DPBS for 20 min at room temperature. TOM20-dHT7-T2A-CalR-HT7-KDEL-expressing cells were fixed using 3% FA and 0.1% GA (Electron Microscopy Science, Hatfield, Pennsylvania) in 1× DPBS (Gibco, Billings, Montana, 14190094) for 20 min at 37°C. For single-color imaging, one of two xHTL, SiR-Hy4 targeting dHT7 on mitochondria and JF_635_-S5 targeting HT7 on vimentin or the ER, was added to the cell sample at a final concentration of 5, 10, or 15 nM in 1× DPBS for high-density HT-PAINT and 500 pM or 1 nM for ground truth (GT) measurements. To perform two-target imaging, SiR-Hy4 targeting dHT7 was added to the cell sample at a final concentration of 5 nM in 1× DPBS to image mitochondria. After the first imaging round, cells were washed with 1× DPBS three times, 5 min each. Then, JF_635_-S5 targeting HT7 was added to the cell sample at a final concentration of 10 nM in 1× DPBS to image the ER.

For live-cell imaging, before imaging, cells were washed once with a prewarmed live-cell imaging solution (Thermo Fisher Scientific, Waltham, Massachusetts). JF_635_-S5 or SiR-Hy4 was added to the prewarmed live-cell imaging solution at a final concentration of 5–15 nM. After an incubation time of 10 min, imaging was carried out on an N-STORM microscope (Nikon, Tokyo, Japan) equipped with an oil immersion objective (Apo, 100×, NA 1.49) and an EMCCD camera (DU-897U-CS0-#BV, Andor Technology, Belfast, UK). Fluorophores were excited with a collimated 647 nm laser beam at an intensity of 0.71 kW/cm^2^ (measured at the objective lens) for fixed cells and 0.32 kW/cm^2^ for live cells at highly inclined and laminated optical sheet (HILO) mode.

PAINT data were acquired with an integration time of 150 ms in fixed cells and 50 ms in live cells using active frame transfer mode with an EM gain of 50, a preamp gain of 1, a readout rate of 5 MHz, and an effective pixel size of 157 nm. The PAINT image was reconstructed from 400 to 25,000 frames depending on the experiment. NIS Elements (Nikon, Tokyo, Japan), LCControl (Agilent, Santa Clara, California), and Micro-Manager (34) were used for setup control and data acquisition. For live-cell measurements, an uno stage top incubator (Okolab, Campania, Italy) was used to keep the cells at 5% CO_2_ and 37°C.

### Image reconstruction

Super-resolution images that served as GT were generated with the localization software Picasso (35). Localization of single molecules was performed using the Localize module of Picasso. Single molecules in each frame were identified with maximum likelihood estimation for integrated Gaussian parameters. Postprocessing was performed using the Filter and Render modules of Picasso. Lateral drift was corrected using the redundant cross-correlation function. Localizations were filtered for the width and height of the point spread function (sx, sy) and the localization precision (lpx, lpy). Localizations appearing in consecutive frames from the same fluorophore were linked within a radius of four times the nearest-neighbor-based analysis localization precision and a maximum dark time of five consecutive frames (36).

### Preparation of training data

To train the DeepSTORM model, a high-density emitter data set with precisely known emitter localizations is required. This high-density data with overlapping PSFs was generated by summing up the low-density experimental PAINT data. A low-density data set was obtained by measuring the U-2 OS-vimentin-HT7 stable cell line with 100 nM JF_635_-S5 in 1× DPBS. The measured data were localized using Picasso, and a localization list was obtained. To remove background signals, the localizations lists were filtered out according to localization precision (lpx, lpy). The measured data with a mean density of 0.109 emitters/*μ*m^2^ were used to generate high-density training patches. Randomly selected patches from the low-density data were summed to generate 30,000 high-density training patches with a mean density of 1.3 emitters/*μ*m^2^ (17 px × 17 px) and, together with the corresponding localization lists, were used for model training. Summing of frames was done using a script available at https://github.com/HeilemannLab/ImageSumming (ImageSumming version 1.0.0). The background value of high-density training patches was adjusted to match the background of experimental high-density patches.

### Training and prediction of the NN

Training and prediction using DeepSTORM 2D was performed on the ZeroCostDL4Mic platform (37) using Google Colab cloud computing resources. For the NN training we utilized a single Tesla T4 GPU (CUDA version 12.0 and Tensorflow version 2.12.0). We loaded the 30,000 artificially summed training patches into the DeepSTORM notebook, where they were cropped to 16 px × 16 px and set the number of patches per frame to 1. The upsampling factor was chosen as 16 for fixed cells and 8 for live cells, which resulted in predicted images with a pixel size of 10 and 20 nm, respectively. The different upsampling factors accounted for higher noise levels in live-cell microscopy data. The NN was trained with a batch size of 256, number of epochs of 100, 15% validation split, and an initial learning rate of 10^−5^.

The lateral drift of experimental high-density raw frames were corrected using the NanoJ-Core plugin (38) available in Fiji (39) before prediction with the trained model.

For prediction, a batch size of 1 was used and the threshold was adjusted by comparing the total localization numbers of predicted images and GT. To predict cellular structures in fixed cells, the model trained with an upsampling factor 16 was used, and, for live-cell measurements, the model trained with upsampling factor 8 was used. Training and prediction parameters are detailed in Tables S1 and S2. A neighborhood size of 3 with activated local averaging was used. After prediction, a super-resolution image and the localization lists can be extracted via postprocessing of the notebook. The localization list was converted into a Picasso-compatible file format and rendered into super-resolution images using Picasso.

### Image quality assessment

To determine the minimum number of frames required to obtain a reasonable quality of predicted images, the resolution was determined and several image similarity metrics were applied to the predicted images obtained with varying frame numbers. The spatial resolution of GT and predicted images was determined using decorrelation analysis (40).

To study the quality of predicted images using image similarity metrics, DeepSTORM images were referenced against GT images. A 2-px Gaussian blur was applied to GT and predicted images. The images were set to 8-bit depth. In GT measurements of mitochondria, a mask was generated for the target structure to compare the similarity between GT and predicted image. Multiscale structural similarity index was calculated using the MS-SSIM Fiji plugin (41). To calculate Pearson’s correlation, structural similarity index (SSIM) (42), mean absolute error, peak signal/noise ratio, and lpips (43), a custom Python script, were used.

### Live-cell movie generation

To generate live-cell movies using the localization lists from high-density HT-PAINT, we developed a custom Python script based on the video generation script published at https://github.com/alonsaguy/DBlink. Using the script, super-resolution images were predicted from batches of 400 frames of the high-density data set using a temporally moving window with a 20 frame overlap between frame batches to maintain continuity of cell dynamics visualization. The video length and the temporal resolution were controlled by varying the window size and the number of overlapping window frames. To suppress highly active fluorophore signals and improve image contrast, the images were saturated according to the 99th percentile and normalized each frame to be in the range [0, 1]. Then, a mean filter was applied to remove localizations related to background noise. Finally, the localization maps were convolved with a 1.5-px Gaussian kernel to obtain smooth structures in the reconstruction. The final images were then stitched temporally to generate a super-resolution movie of live-cell dynamics (Video S1: 30 fps, 1080 px × 720 px, 600 frames).

Video S1. HT-PAINT imaging of CalR-HT7-KDEL in living U2-OS cells

## Results

### Model training and image prediction

SMLM provides nanometer spatial resolution yet suffers from slow imaging speed, limiting throughput and temporal resolution in live-cell imaging. One strategy to increase the imaging speed is the implementation of NNs that can predict SMLM images from high-molecular-density images, such as DeepSTORM (21) or DECODE (23). The performance of these NNs can be further boosted by implementing exchangeable fluorophore labels (26), which provide a constant fluorescence signal for long imaging times. For example, a previous study demonstrated that DeepSTORM can accelerate DNA-PAINT imaging (25).

**Figure 1:**
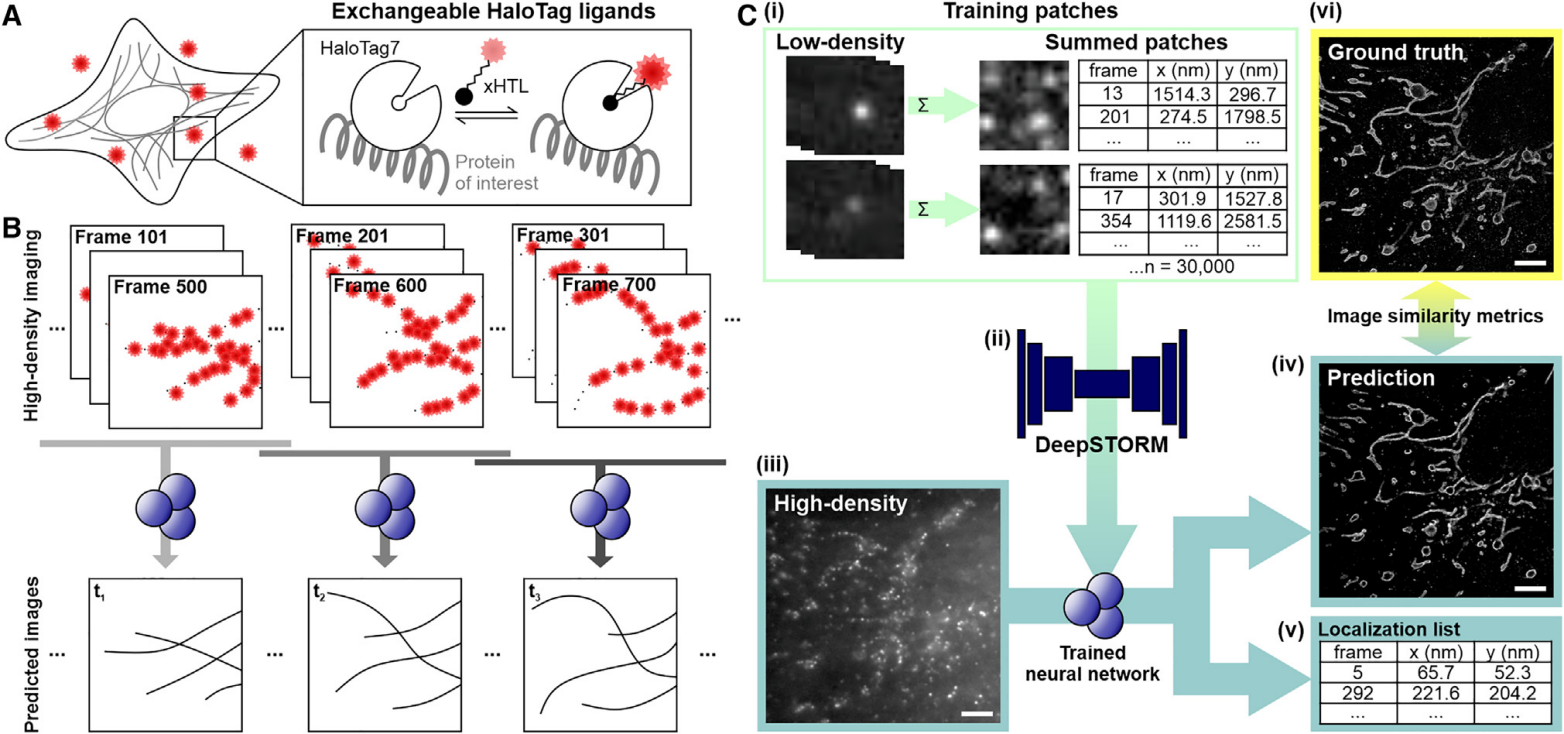
Fast PAINT using HaloTags and exchangeable xHTLs (HT-PAINT) and a neural network. (*A*) Scheme of HT-PAINT in cells. Exchangeable HaloTag ligands (xHTLs) are cell membrane permeable, fluorogenic, and provide a continuous fluorescence signal in PAINT imaging. (*B*) The dynamics of cellular structures can be captured with HT-PAINT. Subsets of frames are used to generate a super-resolved image. (*C*) High-density HT-PAINT prediction with DeepSTORM. (i) Experimental data with sparsely distributed emission events of single molecules are used to generate high-density training patches with precisely known localizations. (ii) Summed high-density patches are used to train a DeepSTORM neural network. (iii) The trained model is used to predict super-resolution images from high-density HT-PAINT data. (iv) DeepSTORM predicts a super-resolution image and (v) the localization lists can be extracted from the postprocessing of DeepSTORM. (vi) To assess the quality of the predicted image, a GT data set with spatially isolated PSFs was measured from the same field of view and structural similarity metrics were used to compare GT and predicted images. Scale bars, 5 *μ*m.

Here, we employ the NN DeepSTORM and use HaloTag7 and exchangeable HaloTag ligands (xHTLs) as direct protein labels (Fig. 1 *A*) (29). We reasoned that high-density imaging with HaloTag7 (HT7) and xHTLs and image prediction with DeepSTORM should reduce the acquisition time. A key feature of xHTLs is that, in combination with fluorogenic rhodamine dyes (44,45), these probes are cell permeable and are applicable to live-cell imaging (29), which should allow their use in visualizing the dynamics of cellular structures in live cells with increased temporal resolution (Fig. 1 *B*).

To predict high-density SMLM data recorded with HaloTag7-labeled proteins (HT-PAINT), we first trained a DeepSTORM NN (21) using experimental training data (Fig. 1 *C*). First, we recorded HT-PAINT data of CRISPR vimentin-HaloTag7 in fixed U-2 OS cells with a sparse emitter density (0.109 emitters/*μ*m^2^) and localized single fluorophore emitters using the Picasso software (35). Small patches (16 px × 16 px) were randomly selected from the low-density experimental data set and summed up to generate high-density training patches (1.3 emitters/*μ*m^2^). The high-density patches, together with the positions of the fluorophores, were used to train a DeepSTORM model. We trained two models with different upsampling factors: for a first model, we applied an upsampling factor of 16 (model 16) to predict structures in fixed cells; for a second model, we applied an upsampling factor of 8 (model 8) to predict structures in live cells. By applying these DeepSTORM models on high-density HT-PAINT data sets, super-resolution images were predicted and localization lists were extracted. To assess the quality of predicted images, these were referenced to a corresponding GT image obtained from low-density imaging of the same field of view applying different structural similarity metrics (Fig. 1 *C*).

### Single model for various high-density SMLM targets

**Figure 2:**
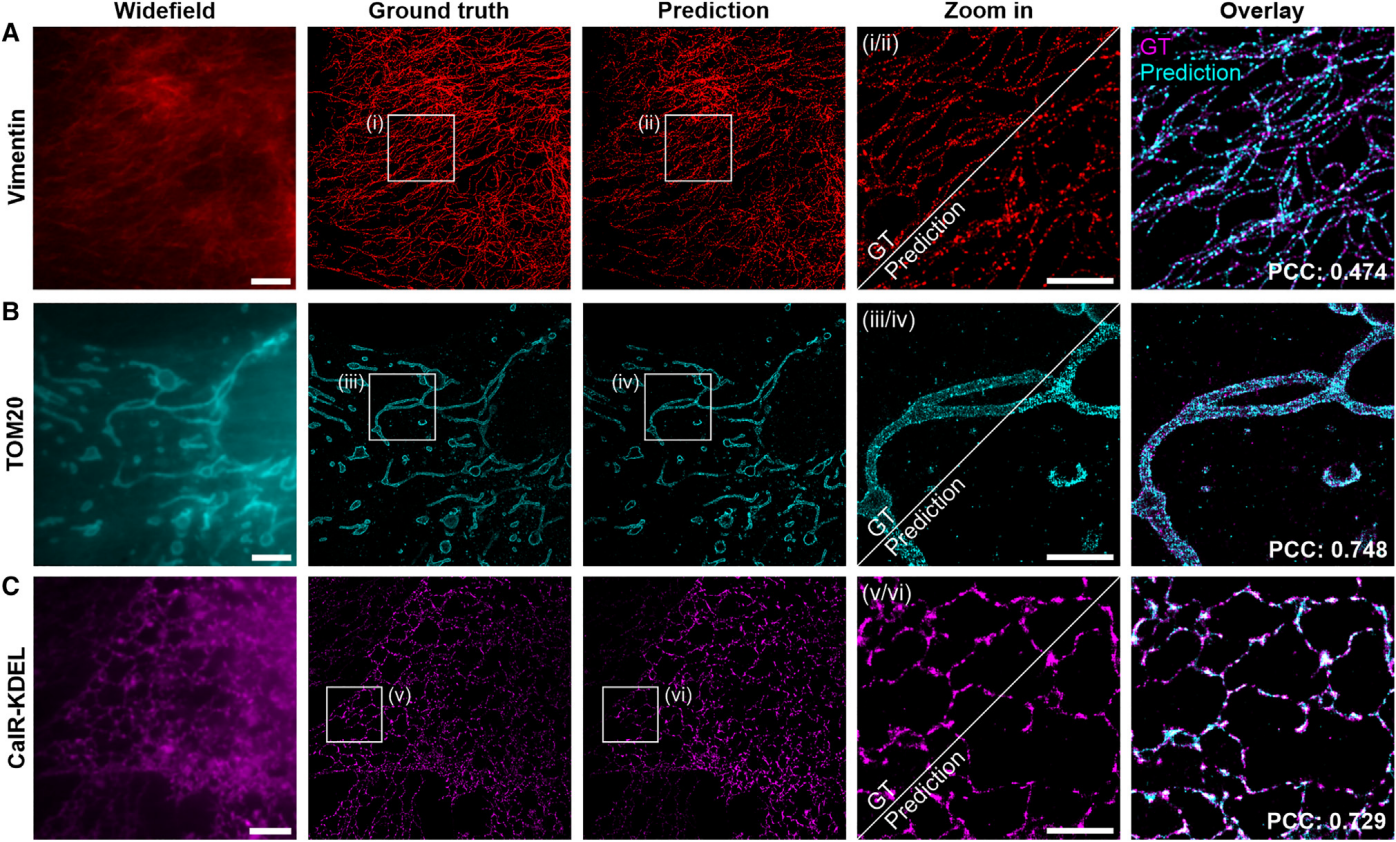
Fast HT-PAINT of cellular structures. (*A*) Vimentin, (*B*) TOM20 (mitochondria), and (*C*) CalR-KDEL (endoplasmic reticulum) imaged in fixed U-2 OS cells. For high-density HT-PAINT data, cells were labeled with 10 nM JF_635_-S5 (*A* and *C*) and 5 nM SiR-Hy4 (*B*). Wide-field images and predicted super-resolution images were generated by the trained DeepSTORM model. For the prediction, high-density movies with 1000 frames were used; numbers indicate spatial resolution as determined through decorrelation analysis. The GT was measured with 1 nM xHTL and localized from the same field of view with 20,000 frames (mitochondria) or 25,000 frames (ER) (vimentin). A single model trained with low-density vimentin data is used to predict mitochondria and ER structures. Zoom-ins (i–vi) are shown for the ground truth and the prediction; overlay images show the similarity of GT and prediction. The Pearson’s correlation coefficients (PCCs) were calculated from the whole field of view. Scale bars, 5 *μ*m (overview) and 2 *μ*m (zoom-in).

The model trained with an upsampling factor of 16 (model 16) was applied to predict SMLM images from high-density HT-PAINT data of three cellular structures, vimentin, TOM20 for mitochondria, and CalR-KDEL for ER (Fig. 2) (the parameters used for training and prediction are listed in Table S1). Cells expressing vimentin-HT7 were labeled with JF_635_-S5. A low-density GT (25,000 frames) and a high-density HT-PAINT data set (1000 frames) were recorded, and a super-resolution image was predicted from the high-density data set (Fig. 2 *A*). Visual inspection shows a good similarity of the filament structure, and a Pearson’s correlation coefficient (PCC) of 0.474 was obtained. The spatial resolution was assessed using decorrelation analysis (40) and yielded 21 nm (GT) and 20 nm (predicted), respectively. HaloTag7 mutant dHT7 fused to a mitochondrial targeting sequence (TOM20) was expressed in U-2 OS cells and labeled with SiR-Hy4, an xHTL that binds preferable to dHT7 (29). Albeit that the SiR-Hy4 ligand was not used for training the NN, the mitochondrial structure was predicted with high structural similarity to the GT, yielding a PCC of 0.748 (Fig. 2 *B*). Decorrelation analysis yielded a spatial resolution of 20 nm (GT) and 30 nm (predicted), respectively. To visualize the ER, cells expressing CalR-HT7-KDEL were labeled with JF_635_-S5, and a PCC of 0.748 was obtained (Fig. 2 *C*). The spatial resolution was 25 nm (GT) and 30 nm (predicted), respectively. These data demonstrate that a single trained model could be applied to predict different cellular structures. Notably, the NN was trained with data recorded for one xHTL only, JF_635_-S5, and could be applied to structures labeled with other xHTLs. We also analyzed the high-density HT-PAINT data with a single-molecule localization software, which was not capable of accurately reconstructing the underlying cellular structures (Fig. S1). In addition, we assessed the image similarity using the MS-SSIM metrics (Fig. S2).

To assess how the quality of the predicted image depends on the number of input frames, various image similarity metrics were used (Figs. S3–S5). For that, the localization lists obtained from a postprocessing step in DeepSTORM were compared with the GT image. The results indicate that, for the conditions tested, 1000 input frames are sufficient to yield a predicted image with good quality, which translates to a 25-fold decrease in acquisition time.

### Two-target and live-cell imaging

**Figure 3:**
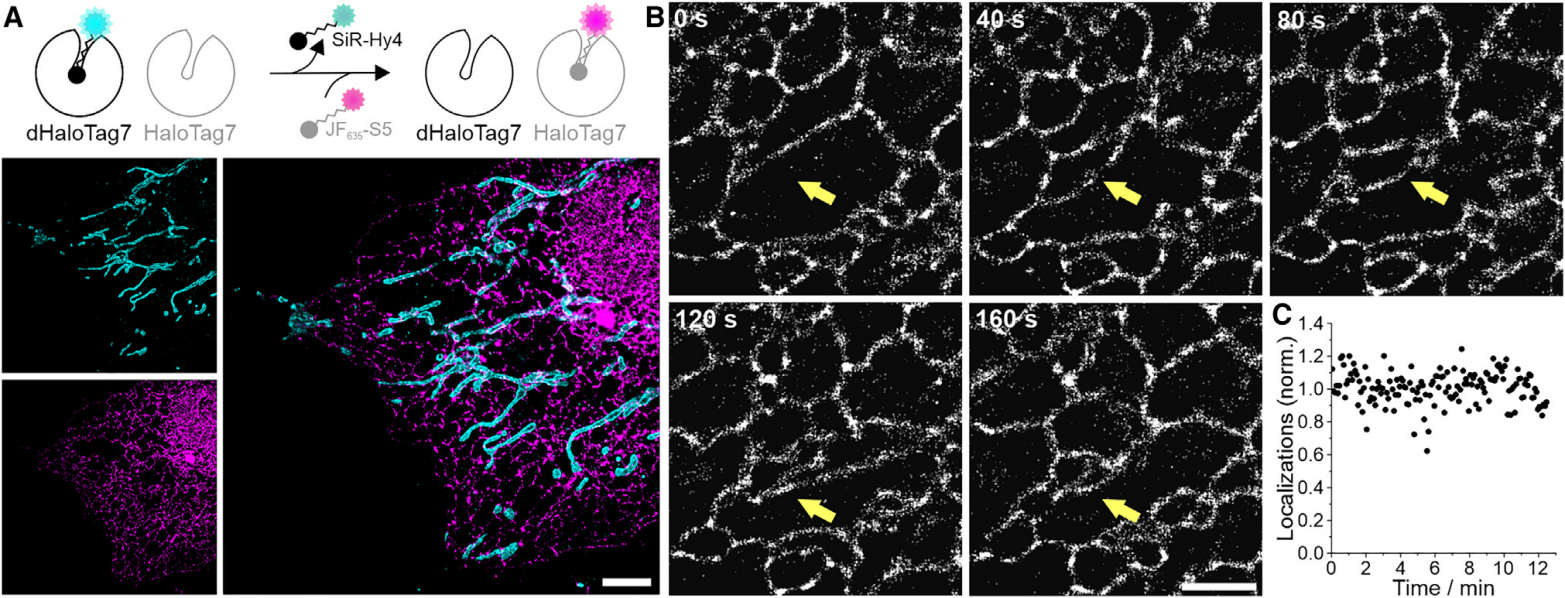
Fast HT-PAINT of two targets and live-cell HT-PAINT. (*A*) Two-target HT-PAINT using orthogonal dHaloTag7 (TOM20, SiR-Hy4) and HaloTag7 (CalR-KDEL, JF_635_-S5) with different xHTL specificity. Super-resolution images from the high-density HT-PAINT measurements were predicted with DeepSTORM. Scale bar, 5 *μ*m. (*B*) Live-cell HT-PAINT of CalR-KDEL-HaloTag7 labeled with JF_635_-S5 recorded for a total time of 12.5 min at an integration time of 50 ms. Single super-resolved images were predicted from a subset of 400 raw image frames. Scale bar, 2 *μ*m. The yellow arrows point to dynamics in the ER structure. (*C*) Fluorescence signal stability during live-cell measurements shown as normalized number of localizations per 100 frames.

The data on single-target structure prediction indicates that the trained model is independent of target morphology. We then applied the model to two different structures to perform dual-target multiplexing with HT-PAINT in the same cell (Fig. 3 *A*). For that purpose, we used a cell expressing two HaloTag7 mutants, HT7 and dHT7, that differ more than 50-fold in their affinity for different exchangeable ligands and allow selective two-target imaging in cells (29). In the first step, we performed HT-PAINT of dHT7-tagged TOM20 labeled with SiR-Hy4. In the second step, and after washing out the first xHTL, CalR-HT7-KDEL was labeled with JF_635_-S5 and imaged. The DeepSTORM NN was applied to these high-density data, and two-target super-resolution images were generated by aligning the predicted images.

A key advantage of HaloTag7 and xHTL ligands is their cell permeability and the possibility of live-cell labeling (29). We reasoned that HT-PAINT data recorded in live cells should show an equal reduction in the image acquisition time when used in conjunction with DeepSTORM, which would increase the temporal resolution. In addition, exchangeable xHTL should enable observations for extended time periods without signal loss, while their fluorogenicity suppresses background intensity.

To test this assumption, we recorded high-density HT-PAINT data from a live cell expressing HT7-tagged CalR-KDEL (ER) that was labeled with JF_635_-S5 (Fig. 3 *B*; Video S1). A total of 15,000 frames was recorded (12.5 min) and analyzed with the model trained with an upsampling factor of 8 (model 8), which accounted for an increase in noise observed in live-cell imaging data with a short integration time of 50 ms (see materials and methods). The parameters used for training and prediction can be found in Table S2. Single super-resolved images were predicted from 400 input frames. We applied a sliding window and generated predicted images at a step size of 20 input frames (see materials and methods), yielding a video of super-resolved snapshots with a temporal spacing of 1 s showing the dynamics of the ER in a live cell (Fig. 3 *B*; Video S1). Notably, the fluorescence signal remained constant for the entire duration of the live-cell imaging experiment (Fig. 3 *C*), confirming that exchangeable xHTLs are efficient “renewable” target labels for live-cell microscopy.

## Discussion

Although SMLM achieves nanometer spatial resolution, it suffers from slow imaging speed. Recently, deep learning approaches such as DECODE (23), DeepSTORM 2D (21), DeepSTORM 3D (21,22), and ANNA-PALM (19) were developed to overcome this limitation and to accelerate data acquisition for SMLM.

In this article, we introduce a direct protein tag, HaloTag7, in combination with weak-affinity and cell-permeable ligands (29), which enable fast high-density imaging in fixed and living cells. Using our approach, we were able to reduce imaging time for various cellular structures without sacrificing the spatial resolution. This is comparable with previous reports that used NNs for image prediction from high-density imaging data: for the DECODE network, an acceleration by one order of magnitude was reported (23), and DeepSTORM in combination with DNA-PAINT achieves similar performance (25).

For fixed-cell imaging, the acceleration of SMLM data acquisition enhances the experimental throughput, both in numbers of samples and sample size (21,23,25). In addition, live-cell SMLM would profit from such an increase in imaging speed, as it would increase the temporal resolution. However, SMLM with covalent fluorophore labels imposes another limitation to live-cell imaging, since the total observation time would be limited by photobleaching. A solution here is to employ live-cell compatible exchangeable fluorophore labels, which “renew” the fluorescence signal (26), and which were already shown to allow prolonged observation times in live-cell STED microscopy (27,30). In this article, the availability of a live-cell compatible protein tag, HaloTag7, in combination with exchangeable fluorophore labels, xHTLs (29), allowed for high-density SMLM (HT-PAINT) imaging in living cells for extended observation times and without detectable loss of signal. Compared with DNA-PAINT (25), HT-PAINT shows similar performance in terms of acceleration and spatial resolution; interestingly, smaller differences for different xHTL concentrations were observed (Figs. S3–S5). Taken together, high-density HT-PAINT resulted in a temporal resolution of seconds, while almost matching the spatial resolution of HT-PAINT in fixed cells. This demonstrates that the dynamics of cellular structures are traceable with SMLM methods by using a combination of weak-affinity fluorophore labels and high-density imaging, disentangling the so-far discussed interdependency of spatial and temporal resolution for SMLM (46,47).

Increasing the acquisition speed in DNA-PAINT can also be achieved by modulating the binding kinetics of DNA hybridization (16,18). High-density HT-PAINT can match and, in some situations, exceed the imaging speed achieved by these efforts; in addition, it brings in the accessibility to intracellular live-cell imaging, which DNA-PAINT has so far not achieved. A live-cell-compatible variant that would possibly allow high-density imaging is peptide-PAINT (48). Another approach that achieved fast and long-time SMLM of ER dynamics in live cells exploited the shift of the on-off equilibrium of a self-blinking fluorophore with polarity (49). This prolonged the observation time, yet still suffered from photobleaching. In this work, we employ renewable fluorophores that bypass photobleaching and ensure a constant fluorescence signal over time (Fig. 3 *C*). The temporal resolution reported by Takakura et al. (49) was achieved by high-speed imaging in combination with high laser intensities, which allowed increasing the readout frequency. In our work, high-density PSF imaging at moderate intensities in combination with a NN to localize overlapping fluorophores, ensures the high temporal resolution.

The implementation of other live-cell labels, e.g., membrane stains (50), protein-specific small-molecule binders (51), or other weak-affinity protein labels that might be tailored for SMLM (52), seem possible. The combination with high-speed image acquisition (53) could further increase the temporal resolution and the throughput. Similarly, other NN-assisted methods for fast super-resolution imaging can be combined with live-cell high-density imaging, e.g., single-frame super-resolution methods that build on fluctuation analysis (20).

In conclusion, the combination of high-density imaging using exchangeable fluorophore labels and image prediction using a NN increases the imaging speed in fixed-cell and live-cell imaging up to 25-fold. The experimental procedure can be implemented on any SMLM microscope, without the need for specific hardware. The DeepSTORM network is accessible through the community effort ZeroCostDL4Mic (37).

## Data and code availability

The raw data is available from the following public repository: https://www.ebi.ac.uk/biostudies/bioimages/studies/S-BIAD864.

## Author contributions

M.H. designed the project. S.J. performed imaging experiments and analyzed and interpreted the data with the help of K.K.N., J.V.R., A.S., M.S.D., and M.H. J.K. generated HaloTag cell lines, and J.K. and K.J. provided reagents. A.S. extended the DeepSTORM software, and A.S. and J.V.R. wrote supplemental code. All authors discussed the results and edited the manuscript.

## Supporting material

Document S1. Figures S1–S5 and Tables S1 and S2

Document S2. Article plus supporting material
