# Supplementary material for: Neural network-assisted single-molecule localization microscopy with a weak-affinity protein tag
Source: Biophys Rep (N Y). 2023 Aug 18;3(3):100123. doi: 10.1016/j.bpr.2023.100123 (PMC10480660; doi:10.1016/j.bpr.2023.100123)
Supplement: Document S1. Figures S1–S5 and Tables S1 and S2 [file mmc1.pdf]

Biophysical Reports, Volume *3*

Supplemental information

Neural network-assisted single-molecule localization microscopy with a weak-affinity protein tag

Soohyen Jang, Kaarjel K. Narayanasamy, Johanna V. Rahm, Alon Saguy, Julian Kompa, Marina S. Dietz, Kai Johnsson, Yoav Shechtman, and Mike Heilemann

Supplemental information

Neural network-assisted single-molecule localization microscopy with a weak-affinity protein tag

Soohyen Jang,[1,2] Kaarjel K. Narayanasamy,[1,3] Johanna V. Rahm,[1] Alon Saguy,[4] Julian Kompa,[5] Marina S. Dietz,[1] Kai Johnsson,[5] Yoav Shechtman[4] and Mike Heilemann[1,2,*]

[1]*Institute of Physical and Theoretical Chemistry, Johann Wolfgang Goethe-University, Max-von-Laue-Str.7, 60438, Frankfurt am Main, Germany*

[2]*Institute of Physical and Theoretical Chemistry, IMPRS on Cellular Biophysics, Johann Wolfgang Goethe-University, Max-von-Laue-Str.7, 60438, Frankfurt am Main, Germany*

[3]*Department of Functional Neuroanatomy, Institute for Anatomy and Cell Biology, Heidelberg University, Heidelberg, Germany*

[4]*Department of Biomedical Engineering, Technion – Israel Institute of Technology, Haifa, 3200003, Israel*

[5]*Department of Chemical Biology, Max Planck Institute for Medical Research, Jahnstr. 29, 69120 Heidelberg, Germany*

* correspondence: heileman@chemie.uni-frankfurt.de

Supplemental figures

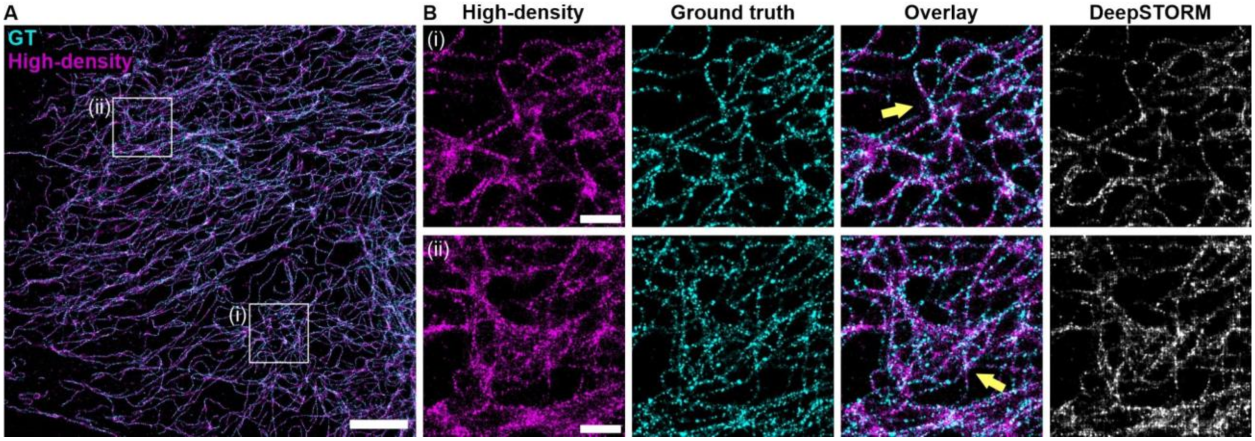

Figure S1. Comparison of ground truth, high-density localization and DeepSTORM prediction of high-density HT-PAINT data. (A) Overlay of high-density HT-PAINT and GT imaging data using the localization algorithm of Picasso (scale bar 5 µm). (B) Zoom-in view of the box in (A) comparing single-molecule localization of high- and low-density (GT) data and DeepSTORM prediction (scale bar 1 µm).

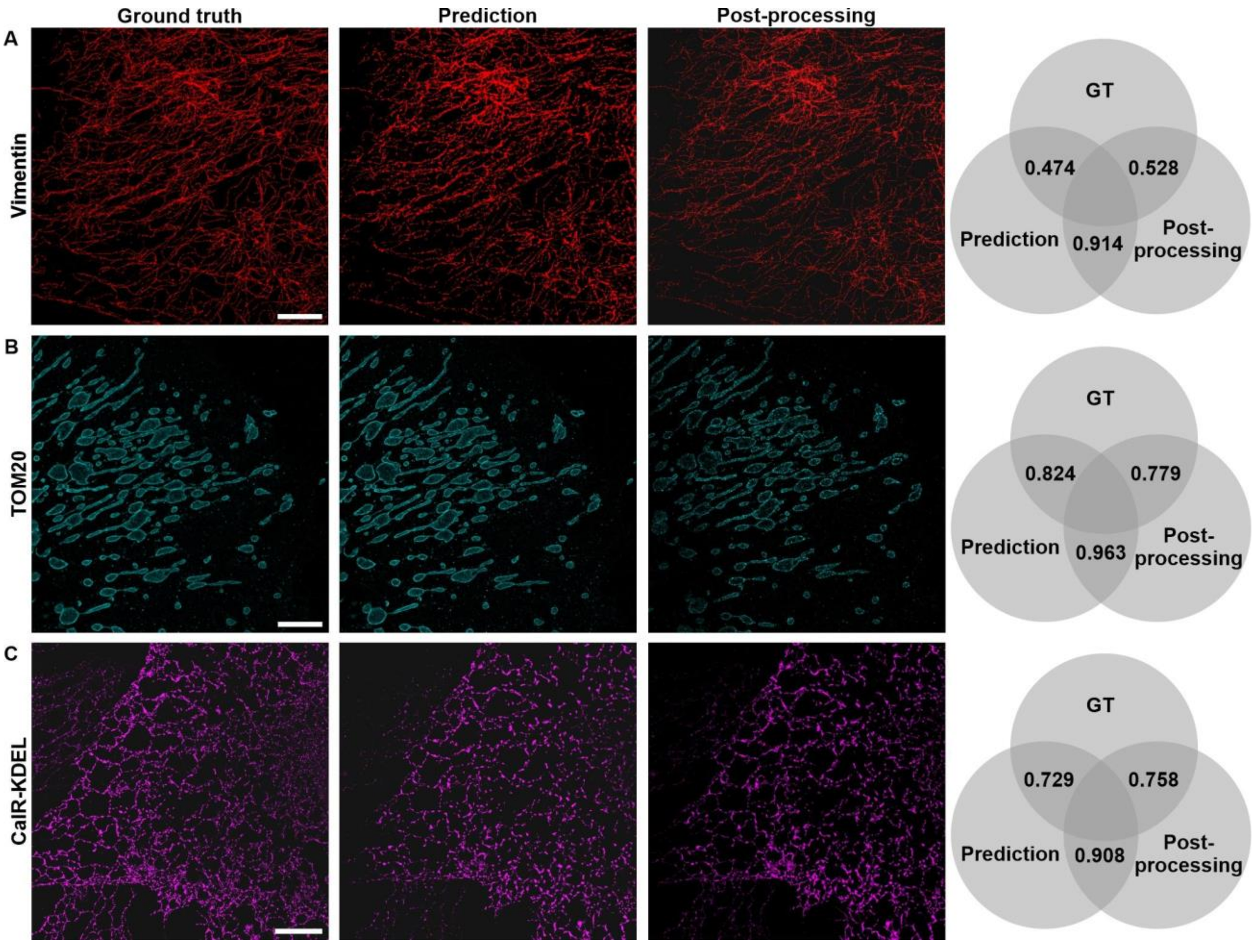

Figure S2. Comparison of the structural similarity of ground truth, predicted and post-processed images. Ground truth, DeepSTORM prediction and post-processed localization images for (A) vimentin, (B) TOM20, and (C) CalR-KDEL were compared using the structural similarity metric MS-SSIM. Scale bars 5 µm.

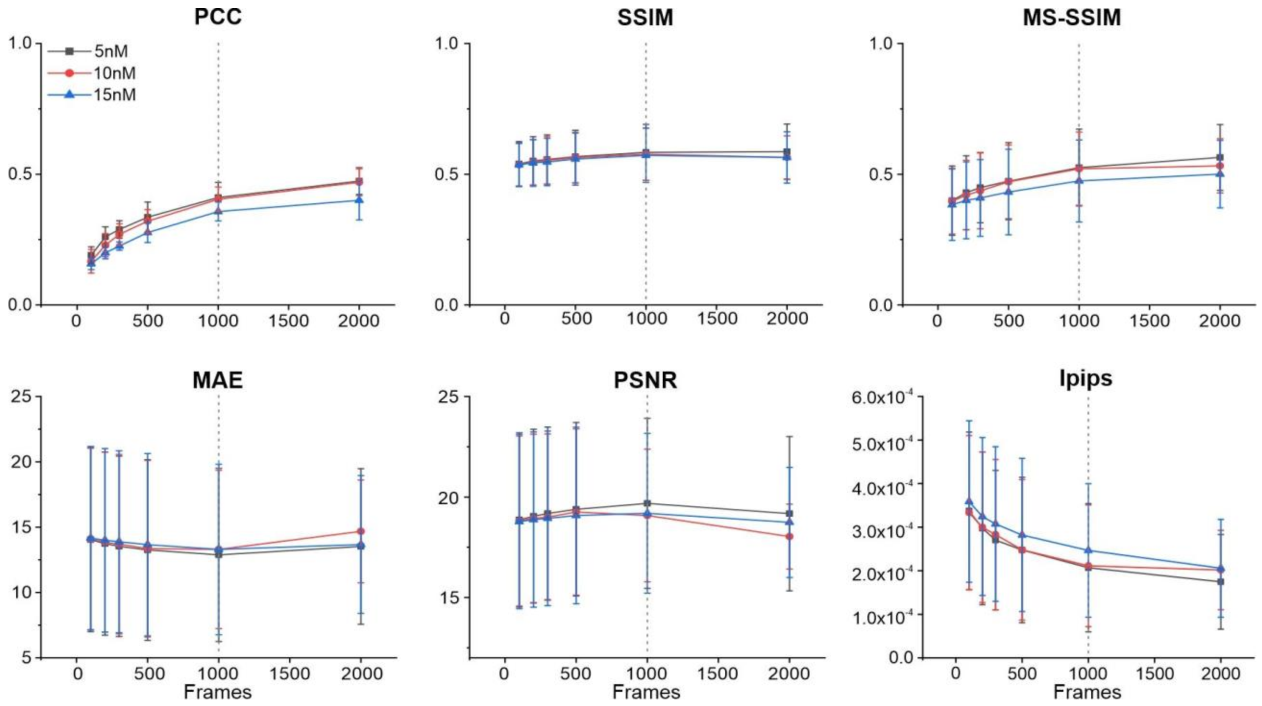

Figure S3. Structural similarity metrics for HT-PAINT data of vimentin. DeepSTORM predictions from high-density HT-PAINT data for various numbers of raw data frames and xHTL concentrations were compared to GT, and image similarity was assessed using Pearson's correlation coefficient (PCC), SSIM, MS-SSIM, mean absolute error (MAE), peak signal to noise ratio (PSNR), and lpips. Error bars represent standard deviations.

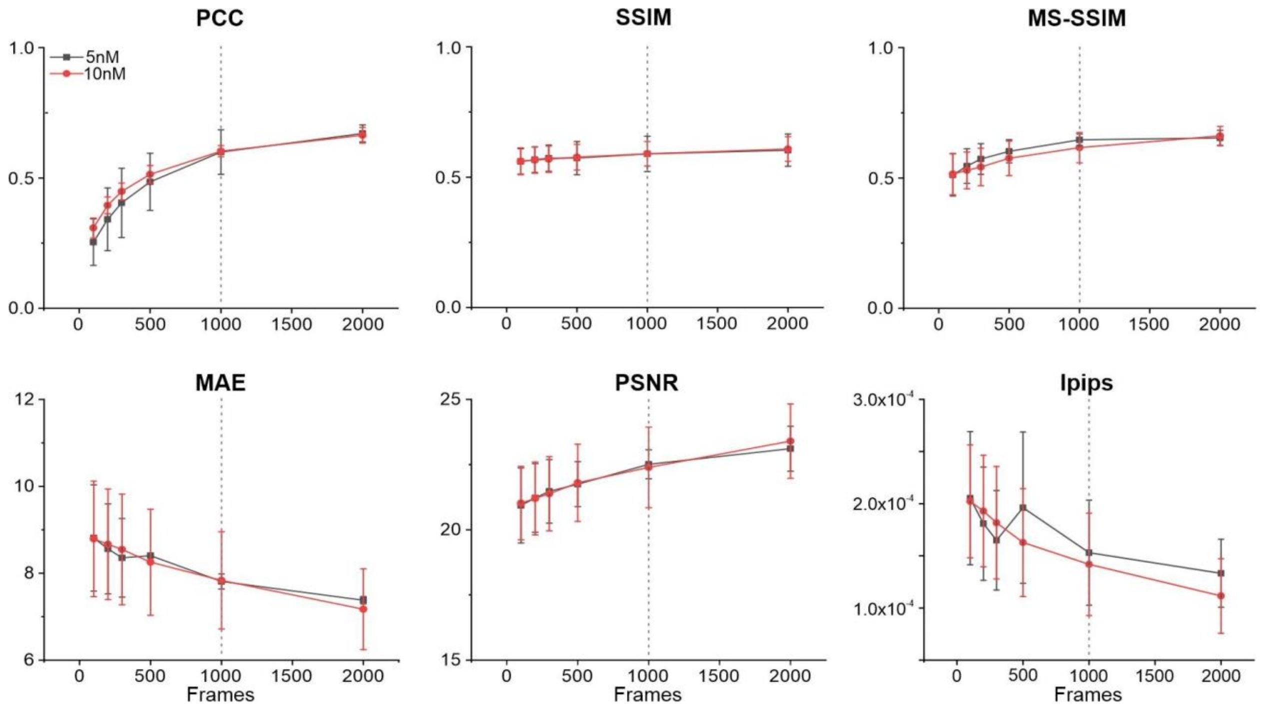

Figure S4. Structural similarity metrics for HT-PAINT data of TOM20. DeepSTORM predictions from high-density HT-PAINT data for various numbers of raw data frames and xHTL concentrations were compared to GT, and image similarity was assessed using Pearson's correlation coefficient (PCC), SSIM, MS-SSIM, mean absolute error (MAE), peak signal to noise ratio (PSNR), and lpips. Error bars represent standard deviations.

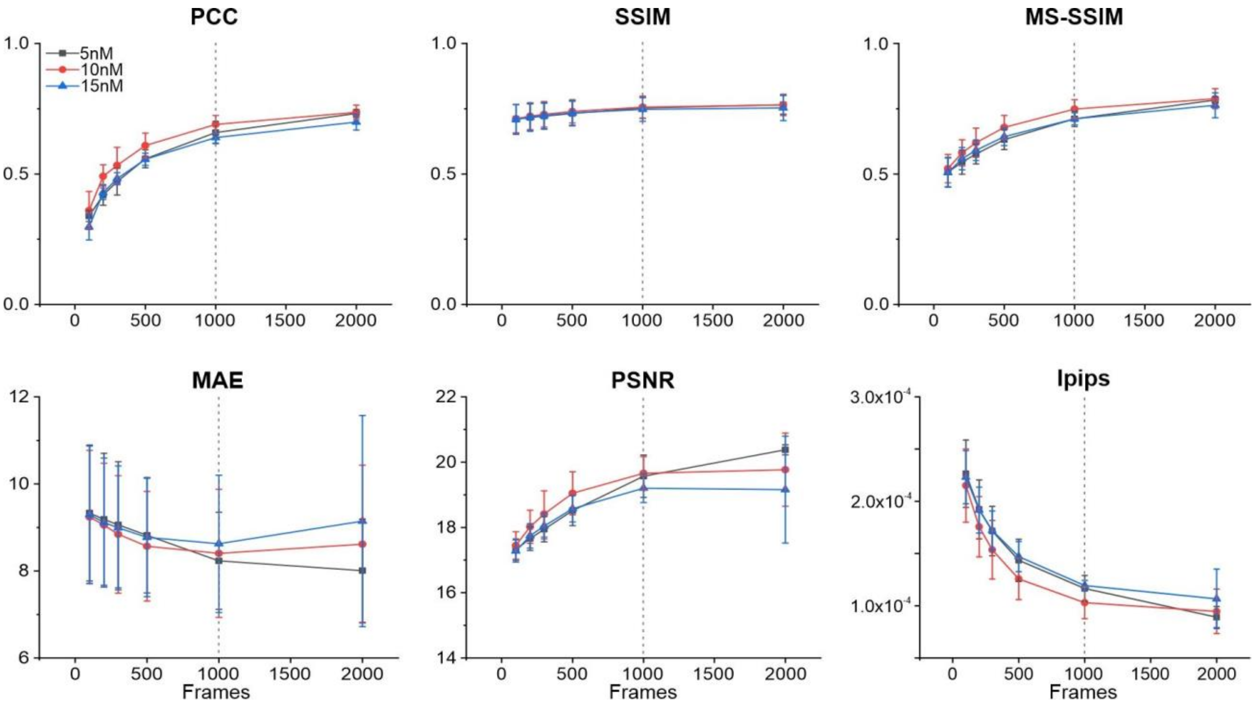

Figure S5. Structural similarity metrics for HT-PAINT data of CalR-KDEL. DeepSTORM predictions from high-density HT-PAINT data for various numbers of raw data frames and xHTL concentrations were compared to GT, and image similarity was assessed using Pearson's correlation coefficient (PCC), SSIM, MS-SSIM, mean absolute error (MAE), peak signal to noise ratio (PSNR), and lpips. Error bars represent standard deviations.

Supplemental tables

Table S1. Training parameters used for training of DeepSTORM model “Model 16”.

**Patch parameter**	
Patch_size	256 x 256
Upsampling_factor	16
Num_patches_per_frame	1
Min_number_of_emitter	1
Max_num_patches	30,000
Gaussian_sigma	1
Automatic normalization	True
**Training parameters**	
Number_of_epoch	100
Batch_size	256
Number_of_steps	99
Percentage_validation	0.15
Initial_learning_rate	1e-05

Table S2. Training parameters used for training of DeepSTORM model “Model 8”.

**Training dataset**		**Patch parameters**	
FOV_size	6400	Patch_size	128 x 128
Pixel_size	160	Upsampling_factor	8
ADC_per_photon_conversion	1	Num_patches_per_frame	500
ReadOutNoise_ADC	15	Min_number_of_emitters_per_patch	1
ACD_offset	100	Max_num_patches	10,000
Emitter_density	2	Gaussian_sigma	1
Emitter_density_std	0	Automatic normalization	True
Number_of_frames	20	**Training parameters**	
Sigma	185	Number_of_epochs	100
Sigma_std	5	Batch_size	32
N_photons	1750	Number_of_steps	234
N_photons_std	250	Percentage_validation	0.25
		Initial_learning_rate	1e-03
